# “I never should have been a doctor”: a qualitative study of imposter phenomenon among internal medicine residents

**DOI:** 10.1186/s12909-022-03982-8

**Published:** 2023-01-24

**Authors:** Alaina Chodoff, Lynae Conyers, Scott Wright, Rachel Levine

**Affiliations:** 1 Division of General Internal Medicine, Greater Baltimore Medical Center, Baltimore, MD USA; 2 Division of General Internal Medicine, University of Michigan, Ann Arbor, MI USA; 3 Division of General Internal Medicine, Johns Hopkins Bayview Medical Center, Johns Hopkins University School of Medicine, Baltimore, MD USA

**Keywords:** Imposter phenomenon, Medical trainees, Medical education, Learning environment

## Abstract

**Introduction:**

Imposter phenomenon is common among medical trainees and may influence learning and professional development. The authors sought to describe imposter phenomenon among internal medicine residents.

**Methods:**

In 2020, using emailed invites we recruited a convenience sample of 28 internal medicine residents from a teaching hospital in Baltimore, Maryland to participate in an exploratory qualitative study. In one-on-one interviews, informants described experiences of imposter phenomenon during residency training. Using thematic analysis to identify meaningful segments of text, the authors developed a coding framework and iteratively identified and refined themes. Informants completed the Clance Imposter Phenomenon Scale.

**Results:**

Informants described feelings and thoughts related to imposter phenomenon, the contexts in which they developed and the impact on learning. Imposter phenomenon has profound effects on residents including: powerful and persistent feelings of inadequacy and habitual comparisons with others. Distinct contexts shaping imposter phenomenon included: changing roles with increasing responsibilities; constant scrutiny; and rigid medical hierarchy. Learning was impacted by inappropriate expectations, difficulty processing feedback, and mental energy diverted to impression management.

**Discussion:**

Internal medicine residents routinely experience imposter phenomenon; these feelings distort residents’ sense of self confidence and competence and may impact learning. Modifiable aspects of the clinical learning environment exacerbate imposter phenomenon and thus can be acted upon to mitigate imposter phenomenon and promote learning among medical trainees.

**Supplementary Information:**

The online version contains supplementary material available at 10.1186/s12909-022-03982-8.

## Introduction

Imposter phenomenon (IP) describes the internal experience of feeling like a fraud and doubting the validity of one’s own achievements [1–3]. IP, common among individuals from diverse demographic and professional backgrounds, is associated with increased rates of depression, anxiety, burnout, and job dissatisfaction [4–7] and may deter individuals from taking risks for career advancement [8]. Although once conceptualized as a phenomenon associated with individual characteristics, such as neuroticism and perfectionism [9, 10], there is growing acceptance that IP is an affective experience rooted in a host of environmental and social contexts [11, 12]. IP seems to be especially common in work cultures that prize precision and results over process, humility, and empathy. [13, 14].

A scoping review among physicians-in-training reveals that IP is prevalent among medical trainees, with rates ranging from 22–60% in medical students and 33–44% in residents [15]. A recent study of over 250 medical students in Canada found that 75% of students met criteria for IP using the Clance Imposter Phenomenon Scale (CIPS) [16]. IP is often associated with self-undermining behaviors such as procrastination, overpreparation, and risk avoidance that may limit personal and professional growth [17–19]. This may be particularly true during residency training where learners experience work environments that include dismissive communication, shaming, and the promotion of productivity over candidness and vulnerability [20]. However, it is useful to make a distinction and note the relationship between IP and other related affective experiences during medical training- such as shame. By comparison shame is a strong emotion that exclusively occurs after negative events and may result in significant psychological distress [21]. While IP feelings may be similarly triggered and exacerbated by a negative outcomes, a key distinctive feature of IP is that it interferes with an individual's ability to recognize their own abilities and objective successes; further IP can be activated by any type of achievement focused task such as presenting a complex patient on rounds or performing a clinical procedure [22]. In their recent perspective piece, Morgenstern and Dallaghan reframe IP as an appropriate situational response to the near constant change, uncertainty and intellectual elitism in the medical profession and suggest that the feelings associated with IP could be an important catalyst for learning, growth, and self-improvement [23].

Although IP is prevalent in medicine, [24–28] there are no qualitative studies that explore IP among post-graduate trainees. In this study, we used qualitative methods to understand how internal medical residents experience IP and its impact on learning.

## Methods

### Study design

We performed a qualitative study using one-on-one, semi-structured interviews to promote safety and comfort among participants who we anticipated may be sharing intense personal stories. At the time of the study, the research team included 2 physicians with medical education research experience including qualitative methodology, 1 recent and 1 current medical education fellow. The interview guide was developed using an iterative process based on published IP literature and the authors’ own experience in GME and with IP [15, 29–31]. Three pilot interviews were conducted to ensure clarity of questions; these interviews led to minor revisions to the final interview guide (Additional File 1). The guide allowed for deeper exploration of concepts and from multiple perspectives as they arose especially around the settings and contexts in which IP arose and its impact. This approach reflects a constructivist paradigm which holds that reality is “constructed” in the mind of the individual and is influenced by specific social contexts and interactions over time [32].

### Setting and participants

A convenience sample of residents from the Johns Hopkins Bayview Internal Medicine residency program in Baltimore, Maryland. All Internal Medicine residents (*N* = 53) were emailed an invitation to participate. We targeted residents in all years of training to capture a breadth of experiences across different learning settings. Twenty-eight residents agreed to participate and received a gift card to a coffee shop for their time.

### Data collection

Interviews were conducted by telephone between May and July of 2020. One author (AC) conducted all interviews which on average lasted approximately 35 min.

At the time of the interviews, AC was a general internal medicine medical education fellow and regularly interacted with residents but did not have a role in resident evaluation. Study informants provided informed consent verbally at the start of the interview. A Johns Hopkins Medicine institutional review board approved the study (IRB00243043).

Limited demographic information including post graduate year (PGY), age, and gender was collected prior to the interview. After each interview, informants were emailed the 20-item CIPS; a validated scale used to measure IP characteristics and severity in an individual. Scores range between 0–100. The higher the total score, the more frequently and seriously an individual is affected by imposter feelings. A score of 60 or above is consistent with frequent imposter feelings [33]. We included the CIPS to demonstrate that our participants experienced imposter feelings consistent with other studies of IP in medicine. We did not use the score to make inferences about individual respondents.

### Data analysis

Audio recordings of the interviews were transcribed verbatim by a professional transcription service and checked for accuracy by the primary researcher/interviewer (AC). Each transcribed interview was uploaded to Computer Assisted Qualitative Data Analysis Software (CAQDAS) for data analysis. We performed thematic analysis using an inductive approach beginning with identifying meaningful segments of text, followed by creating a coding template, categories, and ultimately describing themes [34]. All transcripts were initially read by AC and RL to become familiar with the data and create a provisional coding template. Two additional authors (LC and SW) utilized the provisional coding template to independently analyze four different transcripts each to ensure that most ideas could be captured. An iterative process was used to finalize the coding template. Each transcript was read by at least 3 members of the study team.

The team met multiple times to discuss patterns and relationships from the coded data and name final themes. Illustrative quotes were selected from the original transcripts; the team reached consensus on the best examples to share. Descriptive statistics were used to summarize informant demographics. Mean CIPS scores were calculated.

## Results

Twenty-eight internal medicine residents participated in the study interviews. Table 1 describes informant characteristics. Most informants identified as female (75%), and the mean age was 30 years. The average CIPS score was 63; 16 (57%) informants had a score above the cutoff of 60, suggestive of significant imposter feelings.

**Table 1:** Characteristics and CIPS^*^ scores of informants in a qualitative study of imposter phenomenon during internal medicine training

Characteristic	Total, No. (%)	CIPS Score (mean)
**Gender**	**No. (% of 28)**	
Male	7 (25)	65
Female	21 (75)	63
All informants	28 (100)	63
**Age (years)**	**No. (% of 28**)	
< 30	19 (68)	61
> / = 30	9 (32)	60
**Post graduate year**	**No. (% of 28)**	
First	6 (21)	72
Second	12 (43)	63
Third	10 (36)	56

^*^Clance Imposter Phenomenon Scale (CIPS), scores range 0–100, a score of 60 or above is consistent with frequent imposter feelings

Two categories are presented: (i) lived experience of IP, and (ii) impact of IP on learning, in which themes that demonstrate shared meanings and patterns across the data are identified.

### Lived experience of imposter phenomenon

This category describes the feelings and thoughts that arise when informants were experiencing IP and factors that contribute to IP. For some informants IP was described as intermittent and was associated with isolated events that led to negative self-beliefs. For others, IP was more continuous and pervasive resulting in a constant fear of not “being enough” to succeed in the medical profession. Themes in this category include: (i) *comparisons with others* and (ii) *features of the clinical learning environment.*

### Comparisons with others

Informants described feelings of inadequacy and fraud which were exacerbated by constant comparisons to the performance of others.

Representative quotes are shown below:

“Any small thing that happened - if I missed an order that an intern had put in that maybe shouldn't have happened-- which in the big picture didn’t matter that much, pushed me down the path of – ‘if I can't even do this little thing, how will I be able to do the rest of this. I should be better given that I am this far along.’” (PGY2, Female, Informant #18)

and,

"Sometimes, when I'm working with a second year or third year resident and they are in control of everything and they're really good at what they do, … [I think] ‘oh will I ever be like this?’ I always put a lot of pressure on myself thinking like ‘why am I not like this now?” (PGY1, Male, Informant #26)

### Features of the clinical learning environment

Informants described distinct features of the clinical learning environment that influenced the emergence and perpetuation of imposter feelings, including the setting, timing, and interactions with others. Informants commonly experienced IP during role transitions, often coinciding with increased clinical responsibilities and expectations of greater autonomy. They described fear and anxiety related to the adequacy of their skills and knowledge in adapting to new roles.

“But then you transition from being an intern to a second year and suddenly, only a few days have separated the two experiences, but new interns are turning to you for all the answers.” (PGY3, Female, Informant #4).

and,

“When the nurses come up to you, you have to sound—there needs to be a certain level of confidence that comes out, but it might not match how you're actually feeling.” (PGY3, Female, Informant #2)

This informant describes how the culture of medicine exacerbated IP.

“I started having imposter feelings in medical school. For me, it has to do with position in the hierarchy of medicine. … as a medical student, you're at the bottom. And then intern year, it feels like you're at the bottom again. I do feel like it has to do with the hierarchy and being put in situations where you either feel powerless or you're acutely aware of the hierarchy.” (PGY3, Female, Informant #3)

### Impact of imposter phenomenon on learning

Informants were prone to IP feelings such as intense self-criticism and feelings of inadequacy related to routine aspects of medical training which can support learning and growth. Several themes were identified: (i) inappropriate expectations, (ii) distorted feedback, and (iii) focus on maintaining a façade not on learning.

### Inappropriate expectations

Balancing realistic expectations for their own performance with their role in patient outcomes was a challenge for informants. These two quotes capture a common tendency among trainees experiencing IP to assume sole responsibility for negative or poor patient outcomes.

“We admitted a patient in the ICU with undifferentiated shock and we just couldn't figure out what was going on. I remember feeling very overwhelmed and inadequately prepared. I know I was part of a huge team, but I was very frustrated that I couldn't contribute more. I remember feeling drained, not being able to sleep and feeling like I never should have been a doctor.” (PGY2, Male, Informant #27)

In addition, this quote also demonstrates how IP can influence self-assessment of performance.

“ It's hard...not dwelling on smaller moments but being able to step back and say, ‘like, overall, this is how I'm doing.’ Because no one - we're not robots and I don't think anybody including very seasoned clinicians would do every single step absolutely correctly for every patient.” (PGY2, Female, Informant #6)

Informants also acknowledged how the learning environment fosters inappropriate expectations and IP.

“The way medical education is set up, there is not really much guidance on what kind of information you should know. You are just expected to know everything. And then I think that pressure of needing to know everything, and then taking care of patients and realizing that's not possible, I think that disconnect – at least for me, that was the biggest thing. ...and always feeling like you are underperforming because of that.” (PGY1, Female, Informant #28)

### Distorted feedback

When experiencing IP, informants displayed defeatist thinking and expressed difficulty accepting praise or recognizing personal strengths and successes. They were also less likely to believe and internalize positive feedback.

These representative quotes highlight this theme:

“I hear the feedback that I’m getting, but I really don’t believe it.

Because I feel like people, they're not really telling me the truth about, you know, where I'm at. And yeah, I have feelings of just sort of not measuring up.”

(PGY2, Male, Informant #16)

And,

“So, when I used to feel more like an imposter, if anything happened like a good patient outcome or an achievement, I would always think man, like, how did this happen? I'm not that good. It must have been luck, or it must have been some misunderstanding on the part of whichever person gave me praise.” (PGY3, Female, Informant #1)

This informant describes how feedback with coaching can support growth but acknowledges how IP can interfere.

“So I think part of it is coaching and real feedback, constructive feedback, and direct observation...Granted if you have imposter syndrome, you might not listen to the subjective or objective feedback coming from someone else. But at least you are given the opportunity to say. ‘What do I think about myself?’ and ‘What is this other person observing? Are those things congruent or not?’” (PGY3 Female, Informant #10)

The inability to internalize positive feedback could lead to decreased self-efficacy and self-confidence as is evident in this quote:

"Any time I get positive feedback or any time I'm reflecting, I'm like, ‘I don't know what I'm doing. I shouldn't have been in that position. I shouldn't have been allowed to do that. I don't deserve, you know, any praise.’ For example, I was on the wards a month ago...every time they [interns] would look to me or the attending would look to me or we would be in a really difficult position, I'd be like, ‘why are you looking to me? Like I don't know what I'm talking about. Like I don't know enough of this. I don't have experience. I haven't read enough of everything.’ Even though I'm well aware that after two years I at least know enough to be able to make certain decisions.” (PGY2, Female, Informant #17)

### Focus on maintaining a façade not on learning

Informants described the additional mental and emotional energy needed to manage IP as having a negative impact on learning. They often directed more effort into maintaining a perception of confidence than expressing any vulnerability about their performance that might help to direct growth and learning.

“I really do think that because of the imposter syndrome, and the insecurity that comes with it, I wasn't able to truly focus on learning. I was always more focused on the façade.” (PGY1, Female, Informant #28)

and,

“I think one of the frustrating things for me about imposter syndrome was how throughout intern year I kept getting told, be more confident. Be more confident. And I think just acknowledging that people who are learning a new skill set are not going to be all that confident when they're learning it and that's okay. Like, it's okay to struggle. It's okay to not feel confident. It's okay to not be confident.” (PGY2, Female, Informant #7)

In this quote, an informant describes how anticipating a performance focused situation (starting a new clinical rotation) triggered IP feelings and a need to project competence.

“I remember preparing for that rotation...I felt incredibly triggered...feeling not adequate, not feeling good enough and a lot of anticipation and apprehension...I need to show people that I know what I’m doing. Even though there is no way I can know what I’m doing because I’ve never done that before. I felt like I couldn’t allow myself to feel vulnerable.” (PGY 2 Female, Informant #11)

The informant goes on to say how her senior resident helped her to make the most of the rotation in terms of learning.

"...he very much reframed the whole situation of, like, this is not you proving anything to anyone, this is an opportunity for you to learn." (PGY 2 Female, Informant #11)

This informant describes time lost managing IP that might have been focused on learning.

“I think it [IP] honestly impacted the beginning of every new rotation as a second year so from like the medical ICU, the cardiac ICU, the leukemia service I think it basically made the first couple days of each rotation full of anxiety and fear, when they could have just been full of satisfaction and learning." (PGY1, Female, Informant #1)

## Discussion

To our knowledge, this is the first qualitative study to describe IP among physicians in training. Informants in our study describe how IP led them to be less engaged in learning because they were overly focused on managing IP feelings including fraudulence and low self-confidence. IP further influenced growth and learning in the way that residents interpreted mistakes or constructive feedback (as proof of their inadequacy) as well as accomplishments (as the result of luck). The feeling of being under constant scrutiny and comparisons with peers in a performance focused environment heightened IP.

Prior qualitative studies published on IP exclusively focus on the experience of practicing physicians who describe imposter feelings in situations that they believed could compromise their credibility and reveal self-perceived limitations to their expertise [30, 31]. These findings stress the ongoing impact of IP on core issues of physician identity even after completion of training. Like our study, these qualitive studies of faculty highlight the impact that frequent transitions and hierarchical, performance-focused cultures had in contributing to imposter feelings thus demonstrating that IP begins early in training and may persist in part due to specific types of work environments.

Informants in our study experienced imposter thoughts and feelings in clinical learning environments where the culture valued expertise, hierarchy, and certainty. They also described a feeling of being under constant scrutiny which supplanted a growth orientation and compelled learners towards a “fake it till you make it” or impression management mindset which may have negative impacts on learning [35]. In the business world and more recently in medicine, these types of environments have been described and measured using the concept of psychological safety. Psychological safety is a climate in which individuals feel comfortable taking interpersonal risks like speaking up, asking questions, and acknowledging their own deficits without fear of being judged, shamed, or ignored [36]. By promoting a culture of transparency, curiosity and growth, psychological safety opposes the self-critical and fixed mindsets associated with imposter feelings and instead encourages risk-taking with learning [37]. In studies of the clinical learning environment, residents report increased satisfaction with their training experiences when psychological safety is perceived to be high [38, 39]. Further studies could look for the impact of psychological safety on IP.

Feedback is important for learning, especially in the setting of failure or mistakes but also when learners are performing well. When learners have inappropriate expectations around their performance or role in patient outcomes, high quality, routine feedback especially if combined with coaching may help to mitigate IP by using reflective questioning to explore residents' perspectives on their actions and performance [40]. In the Debriefing with Good Judgment approach described by Rudolph et. al, team members inquire about an individual’s cognitive frames, assumptions, and emotions prior to providing critical judgment about their actions [41]. In this manner, the medical educator serves as a coach, helping to separate a learner’s actions (forgetting to order a medication) from negative thoughts and emotions (“I am a failure”). This approach can potentially interrupt internal assumptions that inhibit learning by flattening the hierarchy and setting up a system of open dialogue and tolerance of intelligent failure. A recent quantitative study exploring self-determination theory and IP suggests associations between medical student orientation toward autonomy, competence, and connectedness in the learning environment with severity of IP [16]. Feedback and coaching may help learners shift from an externally focused achievement orientation to one that is more internally focused on growth. For feedback to be effective, learners also need to accept positive feedback and recognize their own strengths [42]. We know from our study that this is a challenge for learners experiencing IP and will need to be addressed directly. A recent randomized clinical trial demonstrates that coaching can reduce burnout and IP feelings among women residents [43]. Coaching focuses on using skills of inquiry to explore the relationship between an individual's thoughts, feelings, and behaviors; it seeks to help individuals reframe negative thought processes such as doubts about one's abilities and belonging. Coaching skills can be taught to educators and this educational strategy is increasingly used in medical education to support technical skill development and also to address non-technical skills such as trainee professional identify formation and well-being [44, 45].

Several limitations of this study deserve consideration. First, our sampling selected informants who were from a single residency program, the majority of whom identified as female and thus our findings may not be generalizable more broadly. Second, the study subjects volunteered to join this study about IP and so they may have agreed to participate based on having had much stronger experiences with IP. Finally, we can only hypothesize relationships between IP and important medical education concepts such as the learning environment, psychological safety, and feedback. Quantitative and mixed methods studies will be needed to test related hypotheses.

## Conclusions

IP is pervasive within a graduate medical training program and may lead to disengagement from learning. Modifiable aspects of the learning environment such as rigid hierarchy and performance orientation, which if addressed may help to mitigate IP among residents.

## Supplementary Information

**Additional file 1.**

## Data Availability

The datasets used and/or analysed during the current study are available from the corresponding author on reasonable request.

## Abbreviations

IP: Imposter phenomenon
CIPS: Clance Imposter Phenomenon Scale
PGY: Post graduate year
