# Supplementary material for: “I never should have been a doctor”: a qualitative study of imposter phenomenon among internal medicine residents
Source: BMC Med Educ. 2023 Jan 24;23:57. doi: 10.1186/s12909-022-03982-8 (PMC9875476; doi:10.1186/s12909-022-03982-8)
Supplement: Supplementary file 1 — Additional file 1. [file 12909_2022_3982_MOESM1_ESM.docx]

APPENDIX

Interview Guide:

Interviewer: Thank you for taking the time to participate in this interview about Imposter Phenomenon during residency training. I’ll be recording the session. All identifying information will be removed from the transcript. This should last about 30 minutes. Do you have any questions or concerns before we start?

1. Discuss a time or situation during residency training when you felt like an imposter or felt like you might be exposed as a fraud, otherwise known as imposter phenomenon?
2. Can you describe some other circumstances or situations when you felt overwhelmed by feeling like an imposter?
3. How have these feelings toward yourself evolved during residency?
4. How, if at all, have these feelings impacted your residency experience?
5. Please explain any attempts you have made to cope with these feelings.
6. Have you encountered other trainees impacted by imposter phenomenon? If yes, share any experiences you have had.
7. Is there anything else you want to share with us about imposter phenomenon that we haven’t covered?
